# Photo-hydrogen and lipid production from lactate, acetate, butyrate, and sugar manufacturing wastewater with an alternative nitrogen source by *Rhodobacter* sp*.* KKU-PS1

**DOI:** 10.7717/peerj.6653

**Published:** 2019-04-04

**Authors:** Thitirut Assawamongkholsiri, Alissara Reungsang, Sureewan Sittijunda

**Affiliations:** 1 Research and Development of GM Plant & Microbe Detection Laboratory/Biotechnology Research and Development Office, Department of Agriculture, Bangkok, Thailand; 2 Department of Biotechnology/Faculty of Technology/Khon Kaen University, Khon Kaen, Thailand; 3 Research Group for Development of Microbial Hydrogen Production Process from Biomass, Khon Kaen University, Khon Kaen, Thailand; 4 Faculty of Environment and Resource Studies, Mahidol University, Nakhon Pathom, Thailand

**Keywords:** Bio-hydrogen, Microbial lipid, Photo fermentation, Volatile fatty acids, Purple non-sulfur photosynthetic bacteria

## Abstract

Photo-hydrogen and lipid production from individual synthetic volatile fatty acids (VFAs) and sugar manufacturing wastewater (SMW) by *Rhodobacter* sp. KKU-PS1 with sodium glutamate or Aji-L (i.e., waste from the process of crystallizing monosodium glutamate) as a nitrogen source was investigated. Using individual synthetic VFAs, the maximum hydrogen production was achieved with Aji-L as a nitrogen source rather than sodium glutamate. The maximum hydrogen production was 1,727, 754 and 1,353 mL H_2_/L, respectively, using 25 mM of lactate, 40 mM of acetate and 15mM of butyrate as substrates. Under these conditions, lipid was produced in the range of 10.6–16.9% (w/w). Subsequently, photo-hydrogen and lipid production from SMW using Aji-L as nitrogen source was conducted. Maximal hydrogen production and hydrogen yields of 1,672 mL H_2_/L and 1.92 mol H_2_/mol substrate, respectively, were obtained. Additionally, lipid content and lipid production of 21.3% (w/w) and 475 mg lipid/L were achieved. The analysis of the lipid and fatty acid components revealed that triacyglycerol (TAG) and C18:1 methyl ester were the main lipid and fatty acid components, respectively, found in *Rhodobacter* sp. KKU-PS1 cells.

## Introduction

The unsustainability of fossil fuel consumption and the climate change resulting from fossil fuel combustion have stimulated strategies for developing alternative energy sources that are renewable and eco-friendly (Sagnak & Kargi, 2011). Among the biofuels, bio-hydrogen and biodiesel are considered important alternative energy resources (Assawamongkholsiri, Plangklang & Reungsang, 2016; Assawamongkholsiri et al., 2018; Bagy et al., 2014; Carlozzi et al., 2010). The requirement of hydrogen gas worldwide is 50 million tonnes per year with a growth rate of about 10% per year (Winter, 2005). Bio-hydrogen production methods have been investigated considering those for bio-photolysis using green algae and cyanobacteria, dark fermentation using anaerobic bacteria and photofermentation using photosynthetic bacteria (Nikolaidis & Poullikkas, 2017). Photofermentation has a higher hydrogen yield (HY) than dark fermentation (Hallenbeck, 2011). Purple non-sulfur photosynthetic bacteria (PNSB) such as *Rhodobacter sphaeroides* KKU-PS5, *R. sphaeroides* KD131, *R. sphaeroides* O.U. 001, and *Rhodopseudomonas palustris* WP3-5 have the capacity to convert hydrogen from a single organic acid (Assawamongkholsiri, Plangklang & Reungsang, 2016; Assawamongkholsiri & Reungsang, 2015; Laocharoen & Reungsang, 2014) and mixed volatile fatty acids (VFAs), which are the major substrates in dark fermentation effluents (Lo et al., 2011; Uyar et al., 2009; Yang et al., 2012). Moreover, a variety of wastewaters such as brewery wastewater (Hay et al., 2017; Seifert, Waligorska & Laniecki, 2010a), dairy wastewater (Seifert, Waligorska & Laniecki, 2010b), sugar industry wastes (Assawamongkholsiri et al., 2018; Keskin & Hallenbeck, 2012) and effluent from dark fermentation processes (Argun, Kargi & Kapdan, 2008; Ozmihci & Kargi, 2010; Sagnak & Kargi, 2011) can be used by PNSB. The limitations of photofermentation are its low hydrogen production rate and high raw material costs (Levin, Pitt & Love, 2004). For these reasons, the efficiency of photo-hydrogen production has to be improved as well as developing ways to use alternative carbon sources, such as lactate, acetate and butyrate, which can be easily obtained from wastes containing VFAs. Wastewater from sugar manufacturing processes (SMW) is an attractive feedstock for photo-hydrogen production due to its abundance and high VFAs content. In 2017, Thailand produced more than 10 million tonnes of sugar (Office of the Cane and Sugar Board, 2017) and generated approximately 130 × 10^6^ m^3^ of wastewater. The conversion of this wastewater to the value-added products such as hydrogen is very attractive. However, the compositions and concentration of the carbon substrates have the influences on hydrogen production and cell growth through metabolism of organic acids (Lo et al., 2011). Therefore, the effect of a medium containing various VFAs components, and their concentrations on photo-hydrogen fermentation should be investigated.

A nitrogen source influences the nitrogenase enzyme activity, which is a key enzyme for photo-hydrogen production (Assawamongkholsiri & Reungsang, 2015; Budiman & Wu, 2018). Amino acids, especially glutamate, was favorably used as nitrogen source in photo-hydrogen production (Assawamongkholsiri & Reungsang, 2015; Laocharoen & Reungsang, 2014). Glutamate is an excellent organic nitrogen source for photo-hydrogen fermentation. However, it is expensive which increases fermentation costs (Laocharoen & Reungsang, 2014). Furthermore, the waste from glutamic acid processes has environmental impacts and requires expensive waste treatment (Zhang et al., 2012). Thus, photo-hydrogen conversion of organic wastewater and alternative nitrogen sources such as waste from the process of crystallizing monosodium glutamate (Aji-L) sources can reduce the operating costs and chemical oxygen demand (COD) produced. Some PNSB can synthesize fatty acids and accumulate lipids during the late stationary growth phase (Carlozzi et al., 2010). Therefore, biomass can be harvested from the final stage of hydrogen generation process, and lipid can be extracted as a by-product. These lipids are suitable for use as precursors for biodiesel production (Carlozzi et al., 2010; Kim et al., 2013).

In this study, hydrogen and lipid production from the individual synthetic VFAs were investigated. Subsequently, the hydrogen and lipid production from SMW were conducted in order to practically apply the concepts to the real wastewater.

## Materials & Methods

### Photosynthetic bacteria and culture conditions

The PNSB, *Rhodobacter* sp. KKU-PS1 (GenBank accession number KC478552), was isolated from the methane fermentation broth of an Up-flow anaerobic sludge blanket (UASB) reactor, Khon Kaen University (Assawamongkholsiri & Reungsang, 2015). KKU-PS1 was pre-cultured in an enrichment medium containing 15 mM of DL-malic acid as a carbon source and 3 mM sodium glutamate as a nitrogen source. The inoculum was grown at 25.6 °C, with an initial pH 7.0, at 150 rpm and a light intensity of 7,500 lux using a light emitting diode (LED) lamp as previously reported by Assawamongkholsiri & Reungsang (2015). After 48 h of cultivation, the pure cultures were centrifuged at 7,000 rpm for 10 min and the solid fraction was used as an inoculum for bio-hydrogen and microbial lipid production in a batch fermentation.

### Carbon and nitrogen sources

The two types of carbon sources were used in the current study i.e., individual synthetic VFAs (i.e., lactate, acetate and butyrate) and SMW. SMW was collected from the Mitr Phu Viang Sugar Co., Ltd., Khon Kaen, Thailand. The particles were removed from raw SMW by filtering through cotton wool and then refiltered using filter paper (Whatman No.1) before use as a substrate. SMW consisted of (all in g/L), 8.00 total chemical oxygen demand (tCOD), 2.60 total sugar, 0.043 total nitrogen, 3.99 total VFAs (0.78 lactic acid, 1.45 acetic acid, 0.74 butyric acid, 0.92 propionic acid, 0.10 succinic acid) (Assawamongkholsiri et al., 2018). The two nitrogen sources were used i.e., sodium glutamate and Aji-L. Aji-L was obtained from Ajinomoto (Thailand) Co., Ltd., Pathum Thani, Thailand. The raw SMW and Aji-L were preserved at 4 °C until used.

### Hydrogen and lipid production from individual synthetic VFAs and SWM by *Rhodobacter* sp KKU-PS1

**Figure 1:**
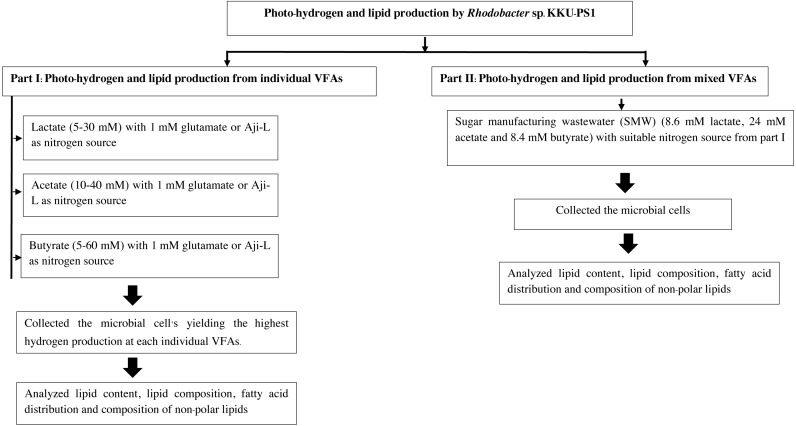
Experimental set up.

This experiment was done in two parts. First, photo-hydrogen and lipid production from individual synthetic VFAs was done in a medium containing various individual synthetic VFAs as carbon sources. Their concentrations were varied in the following manner: lactate (5–30 mM), acetate (10–40 mM) and butyrate (5–60 mM) with either 1 mM glutamate or Aji-L as a nitrogen source. These concentrations were obtained from the literature search (Table S1). The microbial cells from the treatment that yielded the highest hydrogen production were collected to analyze for lipid content, lipid compositions, fatty acid distributions and compositions of non-polar lipids. Second, SMW were used as the carbon source to produce hydrogen with a suitable nitrogen source from the first part of these experiments. After that, microbial cells were harvested to analyze their lipid content, lipid compositions and fatty acid distributions and their compositions of non-polar lipids. The experimental set up is depicted in Fig. 1.

### Experimental set up

Photo batch fermentations were done in 300 mL serum bottles with a working volume of 180 mL. There were four replicates for each experiment. Various combinations of carbon and nitrogen sources were used to supplement the hydrogen production medium (HPM). The HPM consisted of (all in g/L): KH_2_PO_4_, 3.9; K_2_HPO_4_, 2.8; MgSO_4_.7H_2_O, 0.2; CaCl_2_.2H_2_O, 0.075; Na_2_Mo_4_.2H_2_O, 0.01 and 1 mL/L of a stock solution of trace elements (Biebl & Pfennig, 1981). The media, containing various VFAs concentrations and nitrogen sources were sterilized in an autoclave at 121 °C for 15 min. After cooling, 21.7 mL/L of the vitamin solution (Bianchi et al., 2010) and 330 mg/L of a Fe (stock solution of 50 g/L Fe-EDTA complex) (Biebl & Pfennig, 1981) were added using a sterile technique. Then, 10% (v/v) (equivalent to 0.23 g_cell dry weight(CDW)_/L) of a seed inoculum was added to the serum bottles. The initial pH was adjusted to 7.0 (Assawamongkholsiri & Reungsang, 2015) by adding either 0.2 N HCl or NaOH. The serum bottles were purged with Argon gas for 10 min to ensure anaerobic conditions before incubated in an incubator shaker (WIS-10R, Wisd, Laboratory Instrument, Korea) at 25.6 °C, 150 rpm at an illumination intensity of 7500 lux (LED lamps, E27 Corn - 1205, epiStar) (Assawamongkholsiri & Reungsang, 2015).

### Analytical methods

The biomass concentration was determined by measuring the optical density of the culture at a wavelength of 660 nm (OD_660_) using a UV–VIS spectrophotometer (UVmini-1240, Shimadzu, Japan). The CDW were determined from a calibration that showed that 1.0 unit of OD_660_ was equivalent to 0.346 g_CDW_/L (Assawamongkholsiri & Reungsang, 2015). Light intensity was measured using a digital LUX/FC/Light meter (TM-204, TENMARS, Taiwan). The amount of biogas produced was measured by releasing pressure from a serum bottle using wetted glass syringes (Owen et al., 1979). The composition of biogas samples was determined using a gas chromatography (GC) (Shimadzu, GC-2014, Japan) equipped with a thermal conductivity detector. The GC had a stainless-steel column packed with Shin carbon (50/80 mesh, 0.2 × 3 m diameter) and the GC analysis was conducted following the method of Saraphirom & Reungsang (2011). The VFAs concentrations were analyzed using a high-performance liquid chromatograph (HPLC) (LC-20AD, Shimadzu, Japan) equipped with a refractive index (RI) detector and an ultraviolet (UV) detector operated at a wavelength of 210 nm with a VertiSep™ OA 8 µm HPLC column (7.8 mm  × 300 mm). The mobile phase was a 5 mM sulfuric acid solution and at a flow rate of 0.5 mL/min. All soluble samples were prepared prior to HPLC analysis according to Saraphirom & Reungsang (2011). The substrate conversion efficiencies (SCE) were calculated as a percentage by dividing sum of HY from each VFAs by sum of theoretical HY from each VFAs (Eq. (1)). The theoretical HY from each VFAs is presented in Eqs. (2)–(5) (Uyar et al., 2009).

**Figure 2:**
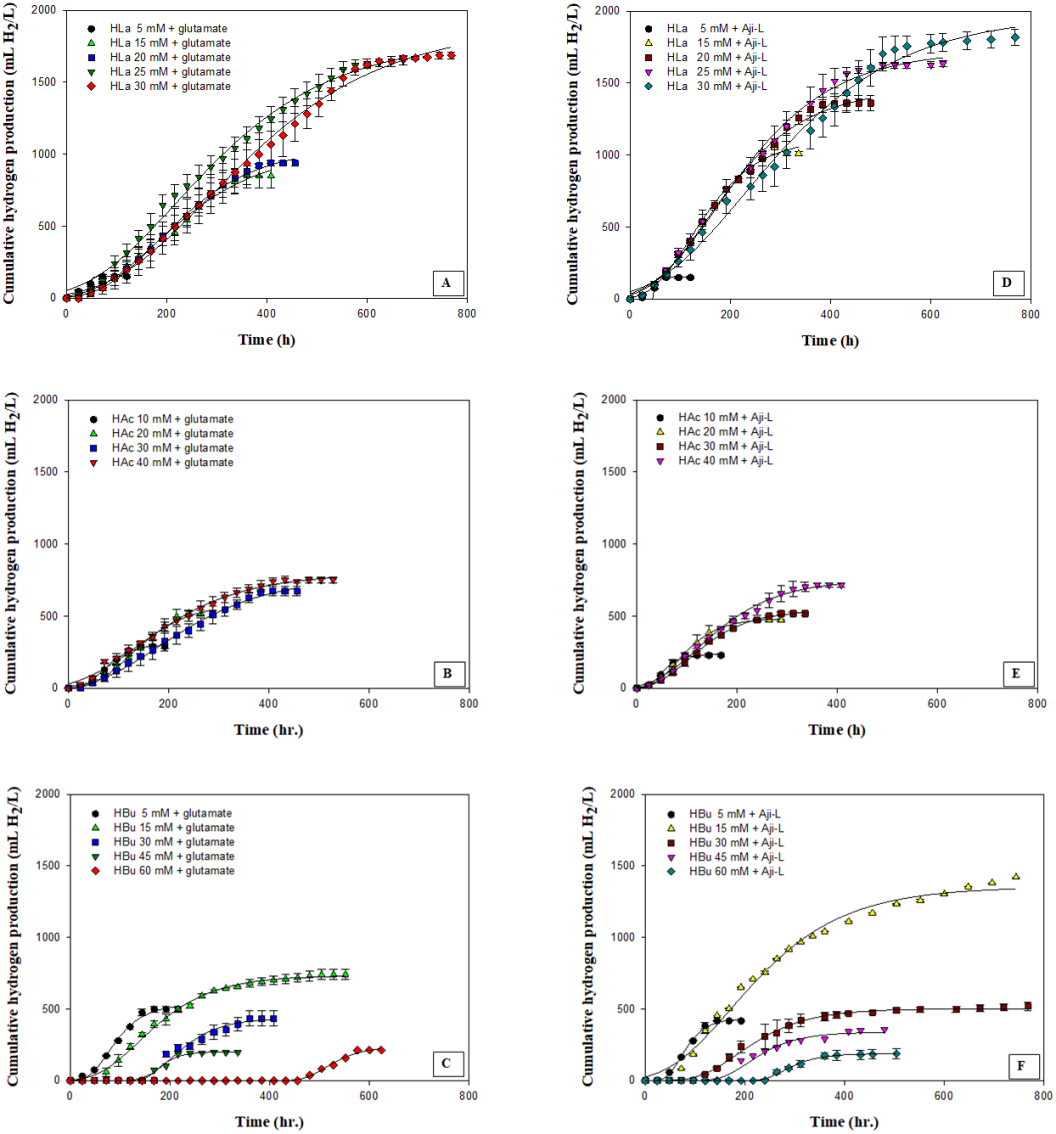
Cumulative hydrogen production using different carbon sources at various concentrations with glutamate or Aji-L as nitrogen sources. (A) lactate (HLa) with glutamate, (B) acetate (HAc) with glutamate, (C) butyrate (Hbu) with glutamate (D) lactate (HLa) with Aji-L, (E) acetate (HAc)with Aji-L, and (F) butyrate (Hbu) with Aji-L.

(1) \begin{eqnarray*}& & \text{SCE}= \frac{[(\text{HY lactate})+(\text{HY acetate})+(\text{HY butyrate})+(\text{HY propionate})]}{[({\text{HY}}_{\text{theoretical}}\text{lactate})+ ({\text{HY}}_{\text{theoretical}}\text{acetate})+ ({\text{HY}}_{\text{theoretical}}\text{butyrate})+({\text{HY}}_{\text{theoretical}}\text{propionate})]} \times 100\end{eqnarray*}

(2) \begin{eqnarray*}& & \mathrm{Lactate}:{\mathrm{C}}_{3}{\mathrm{H}}_{6}{\mathrm{O}}_{3}+{\mathrm{3H}}_{2}\mathrm{O}\rightarrow {\mathrm{6H}}_{2}+{\mathrm{3CO}}_{2}\end{eqnarray*}

(3) \begin{eqnarray*}& & \mathrm{Acetate}:{\mathrm{C}}_{2}{\mathrm{H}}_{4}{\mathrm{O}}_{2}+{\mathrm{2H}}_{2}\mathrm{O}\rightarrow {\mathrm{4H}}_{2}+{\mathrm{2CO}}_{2}\end{eqnarray*}

(4) \begin{eqnarray*}& & \mathrm{Butyrate}:{\mathrm{C}}_{4}{\mathrm{H}}_{8}{\mathrm{O}}_{2}+{\mathrm{6H}}_{2}\mathrm{O}\rightarrow {\mathrm{10H}}_{2}+{\mathrm{4CO}}_{2}\end{eqnarray*}

(5) \begin{eqnarray*}& & \mathrm{Propionate}:{\mathrm{C}}_{3}{\mathrm{H}}_{6}{\mathrm{O}}_{2}+{\mathrm{4H}}_{2}\mathrm{O}\rightarrow {\mathrm{7H}}_{2}+{\mathrm{3CO}}_{2}\end{eqnarray*}

Lipid extraction and lipid content were determined following the modified procedures of Bligh & Dyer (1959) and Shen & Shao (2005). The lipid compositions were determined using thin layer chromatography (TLC) with hexane/diethyl ether/acetic acid (70:30:1 by volume) as the mobile phase (Fuchs et al., 2011; Mangold & Malins, 1960). Fatty acid compositions were analyzed using gas chromatography–mass spectrometry (GC-MS) according to the modified method of Unagul et al. (2007). The modified methods were briefly explained in Assawamongkholsiri, Plangklang & Reungsang (2016).

To determine the maximum hydrogen production potential (H_max_) and lag time, a modified Gompertz equation Eq. (6) was applied to fit the experimental data (Khanal et al., 2004).

(6) \begin{eqnarray*}\mathrm{M}(\mathrm{t})=\mathrm{P}.\exp \nolimits \left\{ -\exp \nolimits \left[ \frac{\mathrm{Rme}}{\mathrm{P}} \left( \lambda -\mathrm{t} \right) +1 \right] \right\} \end{eqnarray*}

where, M, the cumulative hydrogen production (mL H_2_/L); P, the maximum hydrogen production potential (mL H_2_/L); R_m_, the maximal hydrogen production rate (mL H_2_/L h); *λ*, the lag-phase time (h); t, the incubation time (h) and e is 2.71828.

## Results

### Photo hydrogen production from individual synthetic VFAs with glutamate or Aji-L as a nitrogen source

The effect of various carbon sources, i.e., lactate, acetate and butyrate, which are the primary organic acids contained in SMW, on hydrogen production was investigated using glutamate or Aji-L as a nitrogen source by *Rhodobacter* sp. KKU-PS1. Figure 2 shows the cumulative hydrogen production on particular carbon sources at various concentrations. The results showed that *Rhodobacter* sp. KKU-PS1 produced hydrogen using lactate, acetate or butyrate as its sole carbon source. Moreover, *Rhodobacter* sp. KKU-PS1 was highly efficient (100%) to utilize lactate as a substrate (Table 1). Hillmer & Gest (1977), Kim, Ito & Takahashi (1980) and Miyake, Mao & Kawamura (1984) reported that lactate is the prefer organic acid for bio-hydrogen production by *Rhodobacter* sp. When glutamate was used as a nitrogen source, an increase in the lactate concentration from 5 to 25 mM resulted in an increase in the H_max_, R_m_, HY, and SCE (Table 1). However, when the lactate concentration was further increased to 30 mM, the R_m_, HY and SCE decreased to 3.56 mL H_2_/L h, 3.32 mol H_2_/mol_substrate_ and 55.4%, respectively (Table 1). An increase in the concentration of lactate from 25 to 30 mM showed no significant increase in the H_max_ (Fig. 2A). The pattern of hydrogen production and lactate consumption when Aji-L was used as a nitrogen source was similar to that obtained when glutamate was used (Fig. 2D and Table 1). When Aji-L was used as a nitrogen source, an R_m_, HY and SCE of 4.95 mL H_2_/L h, 3.44 mol H_2_/mol_substrate_ and 57.3%, respectively, were achieved with 25 mM lactate.

**Table 1:** H_max_, R_*m*_, HY, SCE, substrate degradation, biomass concentration and final pH for single carbon sources at various concentrations using glutamate or Aji-L as a nitrogen source by *Rhodobacter* sp. KKU-PS1.

Nitrogen source	Carbon source	Concentration (mM)	H_max_^*^ (mL H_2_/L)	R_m_ (mL H_2_/L.h)	HY^*^ (mol H_2_/ mol substrate)	SCE (%)	Substrate degradation (%)	Biomass concentration (gCDW/L)	Biomass production (gCDW/L)	Final pH
		5	151^a^	2.1	3.07^fgh^	51.2	100	0.66	0.43	7.1
		15	1,033^gh^	3.14	3.55^ghi^	59.2	100	0.76	0.53	7.11
	Lactate	20	1,054^hi^	3.49	2.77^efg^	46.1	100	0.82	0.59	7.11
		25	1,797^k^	3.78	3.68^ghij^	61.3	100	0.82	0.59	7.14
		30	1,897^k^	3.56	3.32^ghi^	55.4	100	1.00	0.77	7.18
		10	306^abcd^	3.35	1.99^cde^	49.7	100	0.58	0.35	7.15
Sodium glutamate	Acetate	20	590^ef^	3.22	1.81^bcd^	45.3	100	0.90	0.67	7.23
		30	775^fg^	2.28	1.22^abc^	30.4	100	1.51	1.28	7.73
		40	795^gh^	2.49	1.20^abc^	30	100	1.57	1.34	7.76
	Butyrate	5	532^ef^	5.01	6.05^l^	60.5	100	0.77	0.54	7.21
		15	734^g^	3.27	4.02^ijk^	40.2	60.5	0.80	0.57	7.17
		30	437^de^	3.04	3.35^ghi^	32.6	29.9	0.83	0.60	7.23
		45	200^ab^	2.92	1.21^abc^	12.1	19.1	0.82	0.59	7.25
		60	240^abc^	2.04	1.48^abc^	14.7	15	0.86	0.63	7.3
		5	150^a^	2.08	3.27^fghi^	54.4	100	0.72	0.49	7.16
		15	1,117^i^	5.43	3.70^ghij^	61.7	100	0.79	0.56	7.21
	Lactate	20	1,466^j^	4.91	3.71^hij^	61.8	100	0.84	0.61	7.16
		25	1,727^k^	4.95	3.44^ghi^	57.3	100	0.95	0.72	7.09
		30	1,978^k^	4.14	3.31^ghi^	55.2	100	1.01	0.78	7.12
Waste from the process of glutamate (Aji-L)	Acetate	10	236^abc^	3.99	1.49^abcd^	37.3	100	0.84	0.61	7.24
		20	486^de^	3.8	1.40^abc^	35.1	100	1.09	0.86	7.26
		30	541^ef^	3.01	1.04^ab^	26	100	1.55	1.32	7.66
		40	754^g^	3.12	1.09^abc^	27.3	100	1.91	1.68	7.88
	Butyrate	5	434^cde^	5.7	4.81^k^	48.1	100	0.86	0.63	7.27
		15	1,353^j^	3.98	4.57^jk^	45.7	97.1	1.11	0.88	7.14
		30	502^ef^	2.38	2.37^def^	23.7	35	1.00	0.77	7.19
		45	339^bcde^	2.29	2.14^abc^	21.4	17.5	0.95	0.72	7.05
		60	187^ab^	1.79	1.17^a^	11.7	13.1	0.92	0.69	7.1

**Notes.**

* Difference letters indicated the differences among individual VFAs concentrations and nitrogen sources in column by Scheffe test (*P* <0.05).

H_max_ maximum cumulative hydrogen production R_m_ maximum hydrogen production rate HY hydrogen yield SCE substrate conversion efficiency

Figures 2B and 2E showed the effects of acetate concentration on photo-hydrogen fermentation. The H_max_ increased with increasing acetate concentration from 10 to 40 mM using glutamate or Aji-L as a nitrogen source (Table 1). An increase in acetate concentrations from 10 to 30 mM led to a decrease in the R_m_ from 3.35 to 2.28 mL H_2_/L h and from 3.99 to 3.01 mL H_2_/L h when glutamate or Aji-L was used as a nitrogen source, respectively (Table 1). A further increase in the acetate concentration from 30 to 40 mM slightly improved the R_m_ (Table 1). A suitable acetate concentration of 40 mM with glutamate or Aji-L gave H_max_ values of 795 and 754 mL H_2_/L, respectively. An increase in the acetate concentration resulted in an increase in the cell concentration (Table 1). The highest biomass production of 1.34 and 1.68 g_CDW_/L was attained at 40 mM acetate with glutamate and Aji-L as a nitrogen source, respectively. In addition, the maximum biomass production was attained at 40 mM acetate with 100% substrate degradation. In comparison to lactate and butyrate, HY, and H_max_ obtained from these two carbon sources were greater than when acetate was used as carbon source (Table 1), implying that acetate is used for growth rather than hydrogen production.

The current study shows that a high acetate concentration (40 mM) was not toxic but stimulated the growth of *Rhodobacter* sp. KKU-PS1. Uyar et al. (2009) reported that acetate was primarily utilized for growth of *Rhodobacter sphaeroides* O.U.001 instead of producing hydrogen. The reduction of hydrogen production might be due to a high cell concentration resulting in decreased transmission of light energy (Chen, Lee & Chang, 2006). Moreover, the results found that pH values gradually increased with increasing acetate concentrations (Table 1). A rapid increased in pH values from 7.15 and 7.24 to 7.76 and 7.88 was observed when glutamate and Aji-L were used as nitrogen sources, respectively, at an acetate concentration of 40 mM. High pH values can destabilize nitrogenase activity as well as decrease R_m_ and HY (Yang et al., 2012). Assawamongkholsiri & Reungsang (2015) reported that a pH value of 7.0 was suitable for photohydrogen production by *Rhodobacter* sp. KKU-PS1. Alternatively, non-optimal pH values affected the ionic concentration and inhibited the activity of nitrogenase resulting in low hydrogen production. However, hydrogen production can be enhanced by controlling pH at a suitable level during fermentation (Kim, Kim & Cha, 2012).

**Figure 3:**
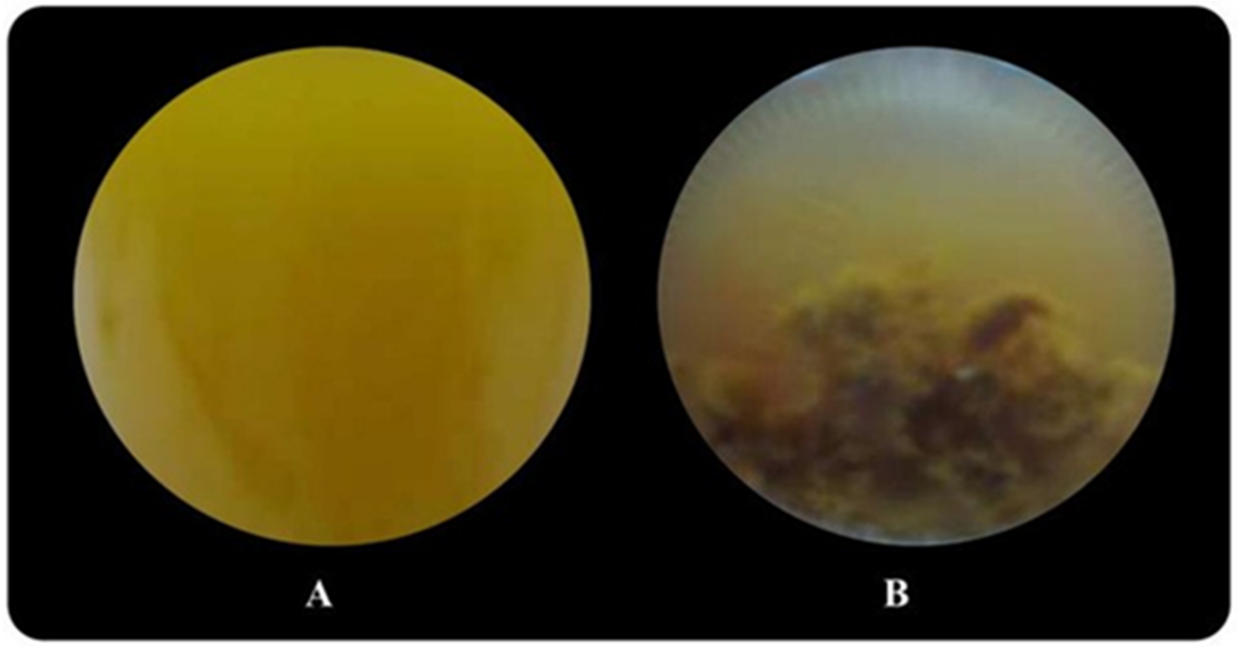
Image of *Rhodobacter* sp. KKU-PS1 in the fermentation broth (A) the live cells in 15 mM butyrate as a carbon source (B) cell death resulted sedimentation in the fermentation broth containing a high butyrate concentration (30–75 mM).

Butyrate was completely consumed at a concentration of 5 mM. The degradation of butyrate decreased to 60.5, 29.9, 19.1 and 15% at 15, 30, 45 and 60 mM, respectively, when glutamate was used as a nitrogen source (Table 1). The results showed that 15 mM butyrate gave the highest H_max_ of 734 mL H_2_/L. Then, it decreased when the butyrate concentration was further increased. Furthermore, when the butyrate concentration was increased to 75 mM, hydrogen production completely stopped (Tables S2 and S3). The pattern of butyrate degradation when Aji-L was used as a nitrogen source was similar to that attained with glutamate (Table 1). However, a higher butyrate degradation, H_max_, R_m_ and HY of 97.1%, 1,353 mL H_2_/L, 3.98 mL H_2_/L h and 4.57 mol H_2_/mol_substrate_, respectively, were achieved at a 15 mM butyrate concentration with Aji-L as a nitrogen source. The results showed that the lag phase and biomass were affected by an increased butyrate concentration. The lag phase increased as butyrate concentration was increased from 5 to 60 mM (Figs. 1C and 1F). The longest lag phase of 456 and 240 h were obtained at 60 mM butyrate with glutamate and Aji-L, respectively, as nitrogen sources. High butyrate concentrations (>30 mM) inhibited hydrogen production. This resulted in lower H_max_, R_m_ and SCE values than those obtained from lactate and acetate as substrates. A similar result was reported by Lo et al. (2011). High butyrate concentrations were toxic to *Rhodobacter* sp. KKU-PS1, leading to decreased cell concentrations, as shown in Table 1. This phenomenon is depicted in Fig. 3. In general, live cells can distribute the limited butyrate contained in a fermentation broth (Fig. 2A). However, excessive butyrate (30–75 mM) can disrupt cells leading to their death and cell sedimentation as shown in Fig. 3B. Loss of cell function may have reduced hydrogen production through substrate inhibition.

The results of the current study indicated that hydrogen production was maximimal at 25 mM lactate, 40 mM acetate and 15 mM butyrate when sodium glutamate or Aji-L was used as a nitrogen source. Various compositions and concentrations of carbon sources affected the kinetics of hydrogen fermentation and the metabolism of cells (Lo et al., 2011). Our results indicate that lactate is the prefer carbon source for hydrogen production by *Rhodobacter* sp. KKU-PS1, while acetate is a suitable carbon source for cell growth. Similar findings were reported by Kim, Ito & Takahashi (1980) and Uyar et al. (2009) who found that lactate was the best substrate for hydrogen production by *Rhodopseudomonas palustirs* while acetate was the best substrate for the growth of *Rhodobacter sphaeroides* O.U. 001. In contrast, butyrate concentrations higher than 30 mM led to a negative effect by decreasing both the hydrogen production and biomass concentration. Normally, lactate is converted to pyruvate and then to acetyl CoA before entering the tricarboxylic acid (TCA) cycle in the metabolism of photosynthetic bacteria. Alternatively, acetate and butyrate do not form pyruvate, but rather are transformed into acetyl CoA (Koku et al., 2002). However, these carbon sources support poly-*β*-hydroxybutyrate (PHB) production rather hydrogen production when the activity of nitrogenase and the TCA cycle is limited (Hustede, Steinbüchel & Schlegel, 1993; Koku et al., 2002).

Using the same carbon source, Aji-L is a more favorable nitrogen source than glutamate for photo-hydrogen production, leading to significantly higher R_m_ values and biomass concentrations as shown in Table 1. Higher cell concentrations led to greater substrate consumption and perhaps enhanced nitrogenase activity of *Rhodobacter* sp. KKU-PS1. Thus, Aji-L is an organic nitrogen source that improved R_m_, biomass concentration and substrate degradation. Glutamate has been reported an excellent nitrogen source for nitrogenase activity in PNSB. However, it is more expensive than inorganic and the other types of nitrogen, which increase photo-hydrogen fermentation costs (Kim, Kim & Cha, 2012; Koku et al., 2002). Aji-L was the best nitrogen source in the current study. Aji-L is rich in glutamate (314 g/L) and consists of ammonium, sulfate and chloride (Assawamongkholsiri et al., 2018). Additionally, it contains reducing sugars, metal ions such as calcium, potassium, manganese, magnesium, phosphorus and iron (Zhang et al., 2012). These are important micronutrients that can support the growth of bacteria and promote nitrogenase activity (Hakobyan, Gabrielyan & Trchounian, 2013).

### Lipid production from individual synthetic VFAs combined with glutamate or Aji-L as a nitrogen source

Lipid was produced as a by-product of hydrogen production. Lipid content and lipid production levels of KKU-PS1 with each optimal single carbon source using glutamate or Aji-L as a nitrogen source are presented in Table 2. With optimal condition for each single carbon source (25 mM lactate, 40 mM acetate and 15 mM butyrate), low lipid content was found, 10.6–16.9% (w/w). Low lipid production was observed, 87–121 mg/L and 98–165 mg/L, when lactate and butyrate was used as substrates, respectively. When acetate was utilized as the sole carbon source, relatively high biomass production (Table 1) and high lipid yield were observed (Table 2). Additionally, a higher lipid production was observed when Aji-L was used as a nitrogen source, rather than glutamate. Acetate and Aji-L were the favored carbon and nitrogen sources to produce high cell concentrations (Table 1). Higher lipid production was supported by enhancing biomass concentration using a photo-heterotrophic cultivation system (Kim et al., 2013).

**Table 2:** Lipid content, lipid production and fatty acid distribution for individual synthetic VFAs under optimal conditions with SMW as carbon sources by *Rhodobacter* sp. KKU-PS1.

Carbon source	Nitrogen source	Lipid content (%, w/w)	Lipid production (mg lipid/L)	Yield (mg lipid/g_CDW_)	Fatty acid distribution (% of total fatty acid)									
					C12:0	C14:0	C15:0	C16:0	C16:1	C17:0	C18:0	C18:1	C:18:2	Other
25 mM lactate	Glutamate	10.6	87	106.10	0.5	0.7	0.1	9.5	3.9	0.7	28.7	52.9	1.4	1.6
25 mM lactate	Aji-L	12.7	121	127.37	0.5	0.5	0.2	7.9	4.1	0.7	29.9	54.4	0.5	1.3
40 mM acetate	Glutamate	14.7	231	147.13	0.8	0.6	0.2	8.9	3.8	0.6	26.1	54.8	2.0	2.2
40 mM acetate	Aji-L	16.9	323	169.11	0.6	0.6	0.2	8.8	3.6	0.7	25.6	56.1	1.7	2.0
15 mM butyrate	Glutamate	12.2	98	122.50	0.5	0.8	0.3	9.0	3.4	2.3	28.2	51.6	1.7	2.3
15 mM butyrate	Aji-L	14.9	165	148.65	0.8	1.3	0.3	11.4	3.4	1.5	26.5	51	1.9	1.9
SMW	Aji-L	21.3	475	213.00	0.7	1.5	1.2	14	3.1	5.7	26.7	43.2	1.3	2.5

**Notes.**

C12:0 lauric acid C14:0 myristic acid C15:0 pentadecylic acid C16:0 palmitic acid C16:1 palmitoleic acid C18:0 stearic acid C18:1 octadecanoic acid C18:2 linoleic acid Other The mixtures of fatty acids, i.e., C19:0 (nonadecylic acid), C20:0 (arachidic acid), C22:0 (behenic acid) and C23:0 (tricosylic acid)

**Figure 4:**
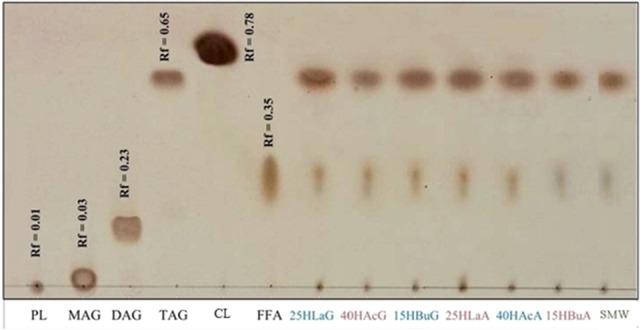
Thin-layer chromatogram of non-polar lipid of *Rhodobacter* sp. KKU-PS1. The reference standards were monoacylglycerols (MAG), diacylglycerols (DAG), triacylglycerols (TAG), cholesteryl linoleate (CL), free fatty acids (FFA) and phospholipids (PL). The lipid band of samples at various conditions were 25 mM lactate with glutamate (25 HLaG), 40 mM acetate with glutamate (40 HAcG), 15 mM butyrate with glutamate (15 HBuG), 25 mM lactate with Aji-L (25 HLaA), 40 mM acetate with Aji-L (40 HAcA), 15 mM butyrate with Aji-L (15 HBuA) and sugar manufacturing wastewater (SMW) with AjiL, respectively.

Crude lipid was extracted from biomass harvested at the end of hydrogen formation. Figure 4 depicts the non-polar lipid fractions of *Rhodobacter* sp. KKU-PS1 on a TLC aluminum silica gel plate. The reference standards for monoacylglycerols (MAG), diacylglycerols (DAG), triacylglycerols (TAG), free fatty acids (FFA), cholesteryl linoleate (CL) and phospholipids (PL) were used to characterize the lipids. TAG was the major component in the lipid accumulated in *Rhodobacter* sp. KKU-PS1, followed by FFA and PL. This lipid pattern was similar at all fermentation conditions, as is shown in Fig. 4. Most bacteria are capable of synthesizing specialized lipids such as polyhydroxyalkanoic acids (PHA) or PHB rather than TAG (Anderson & Dawes, 1990; Kim, Baek & Lee, 2006). However, some bacterial species such as *Rhodococcus opacus* PD360 (Alvarez & Steinbüchel, 2002); (Wältermann et al., 2000) and *Pseudomonas aeruginosa* 44T1 (De Andrés et al., 1991) accumulated TAG as the predominant lipid. In general, PHA is accumulated during the exponential growth phase when the available nitrogen was completely utilized. After this phase, competition between acetyl CoA and propionyl CoA begin. These are the precursors of fatty acids and PHA biosynthesis pathways, respectively. The high level of acetyl CoA is utilized for TAG accumulation rather than PHA synthesis in the late stationary phase (Alvarez & Steinbüchel, 2002; Alvarez, Kalscheuer & Steinbüchel, 2000). In most bacteria, accumulation of TAG and other neutral lipids are usually stimulated when the carbon source in the medium is excessive and the nitrogen source is limited (Alvarez & Steinbüchel, 2002; Ratledge, 2004; Rossi et al., 2011).

The profile of fatty acids and distribution of lipids accumulated by *Rhodobacter* sp. KKU-PS1 under each optimal conditions were investigated (Table 2). The results illustrated that the methyl ester of octadecanoic acid (C18:1) was the primary fatty acid component in the KKU-PS1 strain followed by stearic acid (C18:0), palmitic acid (C16:0), and palmitoleic acid (C16:1), respectively. Normally, fatty acid formation in microorganisms involves a fatty acid synthase (FAS) complex to produce the staring saturated acids (C18) for subsequent desaturation and elongation reactions. A Δ9 desaturase catalyzes the conversion of stearic acid (C18:0) to oleic acid (C18:1n-9) by insertion of the first double bond between carbons 9 and 10 of saturated fatty acid chain (Ratledge, 2004). Lauric acid (C12:0), myristic acid (C14:0), pentadecylic acid (C15:0), margaric acid (C17:0) and linoleic acid (C18:2n-6) were minor fatty acids under all conditions (Table 2). The results showed that although conditions for hydrogen and lipid production were different, the fatty acid distribution and the characteristics of methyl esters generated by KKU-PS1 strain were identical. The percentage of unsaturated fatty acid ranged from 47.6–61.4%, while 37.9–46.6% were saturated fatty acids. The various VFAs and a supplemental alternative nitrogen source (i.e., Aji-L) were efficiently utilized for hydrogen production and generated lipids as by-products. These lipids are primary precursors and are suitable for biodiesel production (Bagy et al., 2014; Kim et al., 2013).

### Photo hydrogen and lipid production from SMW by *Rhodobacter* sp. KKU PS1

**Figure 5:**
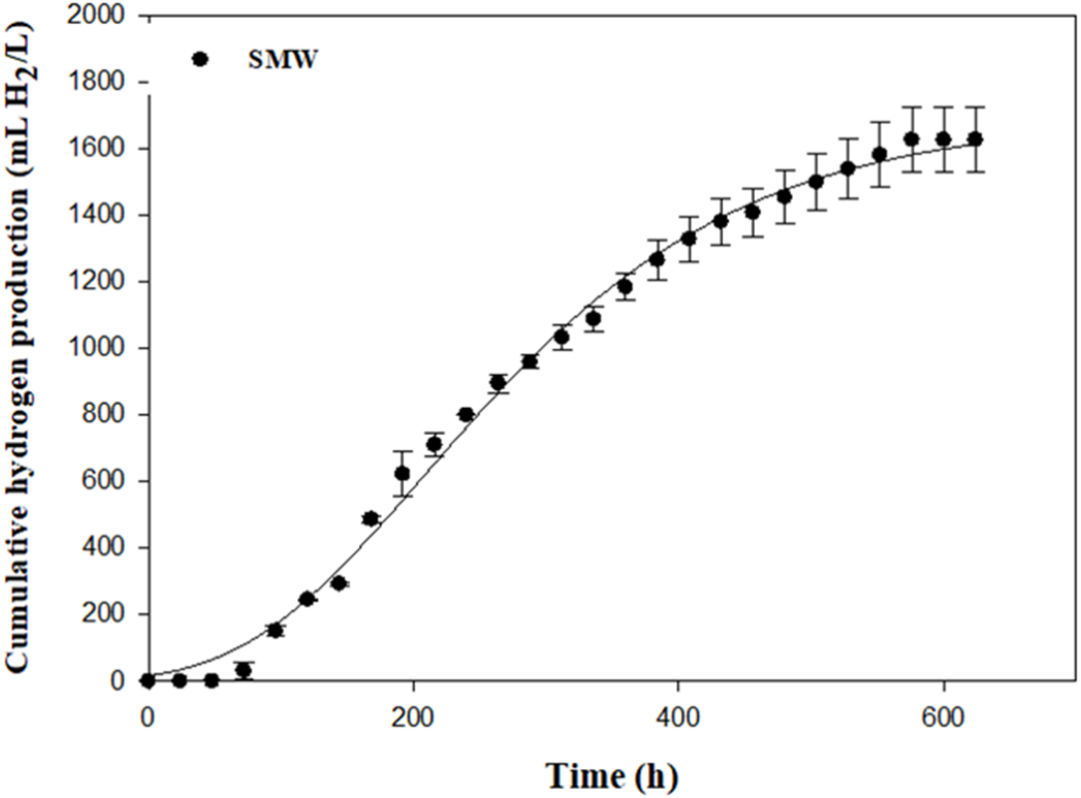
Cumulative hydrogen production from sugar manufacturing wastewater (SMW) with AjiL as a nitrogen source by *Rhodobacter* sp. KKU-PS1.

An optimal R_m_ of 4.62 mL H_2_/L h was found using SMW as carbon source (Table 3 and Fig. 5). SMW contains a mixture of VFAs, including lactic, acetic, propionic and butyric acids that can serve as substrates for photosynthetic bacteria. The results of the current study indicated that *Rhodobacter* sp. KKU-PS1 was highly efficient in production of hydrogen from a wastewater rich in carbon. SMW not only contained lactate, acetate and butyrate but also some sugars, succinate and propionate, as reported in our previous study (Assawamongkholsiri et al., 2018). Thus, the complex chemical compostions and a high initial substrate concentration of 8 g-COD/L influenced the microbial metabolism, which decreased substrate utilization leading to low hydrogen production and HY. The results of the current study were compared to literature values using various organic wastewaters with their individual carbon sources by *Rhodobacter* species (Table 4). Hydrogen production rate and specific hydrogen production rate of 4.62 mL H_2_/L h and 22.00 (mL H_2_/g_CDW_ h) by KKU-PS1 was lower than that of *R*. *sphaeriodes* O.U. 001 (Seifert, Waligorska & Laniecki, 2010a; Seifert, Waligorska & Laniecki, 2010b) (Table 4) but higher than that of *R*. *sphaeriodes* NRRL B-1727 (Ozmihci & Kargi, 2010), *R*. *sphaeriodes* NRLL and *R*. *sphaeriodes* RV (Argun, Kargi & Kapdan, 2008), and *R*. *sphaeriodes* NRRL B-1727 (Sagnak & Kargi, 2011). These results might be attributed to the differences in operation condition, susbstrate type and concentration, as well as microbial species.

**Table 3:** Photo-hydrogen production using SMW with Aji-L as a nitrogen source by *Rhodobacter* sp. KKU-PS1.

Carbon source	H_max_ (mL H_2_/L)	R_m_ (mL H_2_/L h)	HY(mol H_2_/ mol substrate)	SCE (%)	Substrate degradation (%)	Biomass concentration (g_CDW_/L)	Final pH
SMW	1,672 ± 99	4.62 ± 0.08	1.92	7	65.08 ± 0.76	2.23 ± 0.11	7.48 ± 0.01

**Notes.**

H_max_ maximum cumulative hydrogen production R_m_ maximum hydrogen production rate HY hydrogen yield

As shown in Table 4, high hydrogen production rates were reported when mercury-tungsten and halogen lamps were used as light sources. Alternatively, low hydrogen production rates were observed when the light source was fluorescent lamps. High wavelengths (750–950 nm) enhanced the rate of hydrogen production (Argun & Kargi, 2010; Uyar et al., 2007). However, it is difficult to compare literature reports due to differences in substrate composition, bacterial species and cultivation conditions. Non-metabolizable VFAs and lack of a favorable carbon sources might have led to low hydrogen production rates (Ozmihci & Kargi, 2010). Additionally, under a given set of fermentation conditions, various *Rhodobacter* species behave differently due to their differences in metabolic capacities and the variety of VFAs used (Argun, Kargi & Kapdan, 2008). Furthermore, culture conditions critically impact PNSB. Sub-optiaml culture conditions can reduce hydrogen conversion (Assawamongkholsiri & Reungsang, 2015; Kim, Kim & Cha, 2012).

**Table 4:** Comparison of photo-hydrogen production using various organic wastewaters as carbon sources by *Rhodobacter* species.

Feedstock	Substrate concentrations	Microorganism	Operation mode	Light intensity/ type of lamps	Hydrogen production (mL H_2_/L)	Hydrogen production rate (mL H_2_/L.h)	Specific hydrogen production rate (mL H_2_/g _CDW_ h)	Biomass concentration (g_CDW_/L)	Reference
Diluted dairy wastewater	40% of waste (18.5 gCOD/L)	*Rhodobacter sphaeriodes* O.U. 001	Batch fermentation	9 Klux/ mercury-tungsten lamp	3230	49	136.11	2.56^a^	Seifert, Waligorska & Laniecki (2010b)
Diluted brewery wastewater	10% of waste (20.2 gCOD/L)	*Rhodobacter sphaeriodes* O.U. 001	Batch fermentation	9 Klux/ mercury-tungsten lamp	1640	61	169.44	2.28^a^	Seifert, Waligorska & Laniecki (2010a)
Diluted dark fermentation effluent of acid hydrolyed wheat starch	2 g/L of TVFA	*Rhodobacter sphaeriodes* NRRL B-1727	Periodic feeding	5 Klux/ halogen lamps	nd	14.16^a^	8.54^a^	3.64	Sagnak & Kargi (2011)
Diluted dark fermentation effluent of ground wheat starch	2 g/L of TVFA	*Rhodobacter sphaeriodes* NRRL B-1727	Intermittent feeding and effluent removal	5 Klux/ fluorescent lamp	nd	1.13	3.21^a^	0.35	Ozmihci & Kargi (2010)
Diluted dark fermentation effluent of ground wheat	2.68 g/L of TVFA	*Rhodobacter sphaeriodes* NRLL	Batch fermentation	5.5 Klux/ fluorescent lamp	191^a^	1.06^a^	13.80	nd	Argun, Kargi & Kapdan (2008)
Diluted dark fermentation effluent of ground wheat	2.34 g/L of TVFA	*Rhodobacter sphaeriodes* RV	Batch fermentation	5.5 Klux/ fluorescent lamps	126^a^	0.7^a^	3.55	nd	Argun, Kargi & Kapdan (2008)
Sugar manufacturing wastewater (SMW)	4.0 g/L of TVFA (8 gCOD/L)	*Rhodobacter* sp. KKU-PS1	Batch fermentation	7.5 Klux/ LED lamp	1,672	4.62	22.00	2.23	This study

**Notes.**

a the values were calculated from the reported experiment

nd, no reported data

Table 2 and Fig. 4 illustrate the lipid content, lipid production, fatty acid distribution and thin-layer chromatogram of lipids accumulation by *Rhodobacter* sp. KKU-PS1 from fermentation of SMW with Aji-L as a nitrogen source. Maximal lipid content and lipid production of 21.3% (w/w) and 498 mg lipid/L were achieved using SMW (Table 2). Results indicated that the impurities contained in SMW did not inhibit the lipid accumulation of *Rhodobacter* sp. KKU-PS1. Additionally, the maximal lipid content using SMW as the carbon source was 1.68, 1.26 and 1.43 times higher than using 25 mM of lactate, 40 mM of acetate and 15 mM butyrate, respectively, with Aji-L as a nitrogen source. This result might have been due to the higher carbon content of SMW (41 mM of VFAs) than individual synthetic VFAs. A high carbon concentration was favorable for lipid accumulation by *Rhodobacter* sp. KKU-PS1. In our previous study, a carbon excess and nitrogen limited condition (equivalent to a C/N ratio of 132) was suitable for lipid accumulation by KKU-PS1 and gave the higest lipid content and production of 25.4% (w/w) and 592 mg lipid/L, respectively (Assawamongkholsiri, Plangklang & Reungsang, 2016). Kim et al. (2013) reported that a maximum fatty acid productivity of 665 mg fatty acid/L d was attained at lactate concentration of 100 mM. A nitrogen limited condition was found to be an important factor for inducing lipid accumulation in oleaginous microorganisms (Courchesne et al., 2009; Ratledge & Wynn, 2002). The results shown in Fig. 4 indicate that TAG was the major component in the lipid accumulation in *Rhodobacter* sp. KKU-PS1 followed by FFA and PL. The primary methyl esters were octadecanoic acid (C18:1) and stearic acid (C18:0) with yields of 43.2–55.1 and 24.3–26.7%, respectively, with a mixture VFAs as a carbon source. The minor methyl esters were palmitic acid (C16:0), margaric acid (C17:0), palmitoleic acid (C16:1), myristic acid (C14:0), linoleic acid (C18:2n-6), pentadecylic acid (C15:0), and lauric acid (C12:0) (Table 2).

## Discussion

In this study, biohydrogen and lipid production from individual synthetic VFAs and SMW was investigated in the batch experiment. For individual synthetic VFAs, the hydrogen and lipid production by *Rhodobacter* sp. KKU-PS1 depends on types and concentrations of VFAs. The maximum H_max_ was obtained when lactate was used as a carbon source (Table 1) while maximum HY was obtained from butyrate. Kim, Ito & Takahashi (1980) and Miyake, Mao & Kawamura (1984) also found that lactate is a prefer organic acid for bio-hydrogen production. Acetate gave the highest biomass and lipid production of 1.34 g_CDW_/L and 147.13 mg-lipid/g_CDW_ (Tables 1 and 2), suggesting that acetate is suitable for lipid production and the growth of the strain KKU-PS1 but not for hydrogen production. At the highest biomass concentration, the H_max_ and HY was low due to a decrease in light transmission. Thus, the ATP generation via photosynthetic system was reduced. Aji-L is a more favorable nitrogen source than glutamate for hydrogen production as it is rich in glutamate and also contains some trace elements such as magnesisum, iron, and potassium (Zhang et al., 2012). These trace elements are important for enhancing the nitrogenase activity and microbial growth, especially iron. Iron is a cofactor of FeMo-nitrogenase which is the enzyme responsible for hydrogen production by PNSB (Hakobyan, Gabrielyan & Trchounian, 2013).

The H_max_, HY obtained from SMW was lower than from individual synthetic VFAs (Tables 1 and 3) which was caused by the complex compositions and a difficulty to be degraded of SMW. A high initial substrate concentration of SMW, 8 g-COD/L, influenced the microbial metabolism. Lipid and biomass production using SMW as the substrate was higher than using individual synthetic VFAs (Tables 1 and 3). Organic acids such as acetate, succinate, propionate and some sugars contained in SMW are suitable for supporting the growth of microorganisms rather than hydrogen production. The analysis of fatty acid composition by GC-MS found that octadecanoic acid (C18:1) and stearic acid (C18:0) were the main methyl esters accumulated in *Rhodobacter* sp. KKU-PS1. The pattern of fatty acid production from individual synthetic VFAs as the carbon source was similar to the fatty acid production from SMW. The presence of C18:1 and C18:0 as the main fatty acids was correlated to the fatty acid synthase (FAS) system. The FAS system firstly produce the saturated acids (C18) followed by desaturation and elongation reactions (Ratledge, 2004).

## Conclusions

*Rhodobacter* sp. KKU-PS1 produced photo-hydrogen from individual synthetic VFAs (i.e., lactate, acetate and butyrate) and SMW. Additionally, lipid was generated as a by-product during the late stationary phase of photo-hydrogen fermentation. For individual synthetic VFAs, the R_m_ value on lactate was significantly greater than from acetate and butyrate, while the biomass concentration on acetate was higher than from lactate and butyrate. Hydrogen production was maximal at 25 mM lactate, 40 mM acetate and 15 mM butyrate when sodium glutamate or Aji-L was used as a nitrogen source. Lipid production in the range of 10.6–16.9% (of dry biomass) was obtained as a by-product. Aji-L is an excellent alternative organic nitrogen source to increase cell growth, photo-hydrogen and lipid production. For the SMW with Aji-L, maximal hydrogen production and HY of 1,672 ± 99 mL H_2_/L and 1.92 mol H_2_/mol substrate respectively, were obtained. Under this condition using SMW, lipid content and lipid production of 21.3% (w/w) and 475 mg lipid/L, respectively, were achieved. This result indicates the possibility of using *Rhodobacter* sp. KKU-PS1 for integration of hydrogen and lipid (biodiesel) production from alternative carbon and nitrogen sources.

##  Supplemental Information

10.7717/peerj.6653/supp-1 Table S1 Comparison of photo-hydrogen production efficiency of PNSB using various synthetic carbon types and substrate concentrations

10.7717/peerj.6653/supp-2 Table S2 H_max_ ,R_*m*_, HY, SCE, substrate degradation, biomass concentration and final pH for single carbon sources at various concentrations using glutamate as a nitrogen source by *Rhodobacter* sp. KKU-PS1.

10.7717/peerj.6653/supp-3 Table S3 H_max_ ,R_*m*_, HY, SCE, substrate degradation, biomass concentration and final pH for single carbon sources at various concentrations using Aji-L as a nitrogen source by *Rhodobacter* sp. KKU-PS1.

10.7717/peerj.6653/supp-4 Table S4 Photo-hydrogen production using syn-SMW and SMW with Aji-L as a nitrogen source by *Rhodobacter* sp. KKU-PS1

## Additional Information and Declarations

### Data Availability

The following information was supplied regarding data availability: The raw data is available in Tables S1–S4.
