# Supplementary material for: Photo-hydrogen and lipid production from lactate, acetate, butyrate, and sugar manufacturing wastewater with an alternative nitrogen source by Rhodobacter sp. KKU-PS1
Source: PeerJ. 2019 Apr 4;7:e6653. doi: 10.7717/peerj.6653 (PMC6451836; doi:10.7717/peerj.6653)
Supplement: Table S1 [file peerj-07-6653-s001.docx]

| Types of carbon source | Carbon source concentrations (mg/L) | Carbon source concentrations (mM) | Carbon source concentrations (g-COD/L) | PNSB | Substrate conversion efficiency (%) | References |
| --- | --- | --- | --- | --- | --- | --- |
| Lactic acid | 4743 | 52.65 | 5.05 | *Rhodobacter sphaeroides* KD131 | 38 | Kim et al., 2012b |
| Lactic acid | 2072 | 23.00 | 2.21 | Phototrophic Hydrogen-Producing Bacterial Consortium | 8.2 | Lazaro et al., 2012 |
| Lactic acid | 2252 | 25.00 | 2.40 | *Rhodobacter sphaeroides* HJ | 67.7 | Kobayashi et al., 2011 |
| Lactic acid | 2252 | 25.00 | 2.40 | *Rhodobacter sphaeroides* RV | 58.3 | Kobayashi et al., 2011 |
| Lactic acid | 5405 | 60.00 | 5.76 | *Rhodobacter sphaeroides* HY01 | 72.6 | Yang et al., 2014 |
| Acetic acid | 3603 | 60.00 | 3.84 | *Rhodobacter sphaeroides* KD131 | 10.9 | Kim et al., 2012a |
| Acetic acid | 1082 | 18.02 | 1.15 | Phototrophic Hydrogen-Producing Bacterial Consortium | 14.5 | Lazaro et al., 2012 |
| Acetic acid | 1201 | 20.00 | 1.28 | *Rhodovulum sulfidophilum* P5 | 64.6 | Cai and Wang, 2012 |
| Acetic acid | 3603 | 60.00 | 3.84 | *Rhodopseudomonas* sp. nov. strain A7 | 43.9 | Liu et al., 2015 |
| Acetic acid | 1501 | 25.00 | 1.60 | *Rhodobacter sphaeroides* HJ | 62.5 | Kobayashi et al., 2011 |
| Acetic acid | 1501 | 25.00 | 1.60 | *Rhodobacter sphaeroides* RV | 35 | Kobayashi et al., 2011 |
| Butyric acid | 2643 | 30.00 | 4.80 | *Rhodobacter sphaeoides* KD131 | 12.3 | Kim et al., 2012a |
| Butyric acid | 1498 | 17.00 | 2.72 | Phototrophic Hydrogen-Producing Bacterial Consortium | 13.8 | Lazaro et al., 2012 |
| Butyric acid | 1762 | 20.00 | 3.20 | *Rhodovulum sulfidophilum* P5 | 41.8 | Cai and Wang, 2012 |
| Butyric acid | 3524 | 40.00 | 6.40 | *Rhodobacter sphaeroides* HY01 | 50.5 | Yang et al., 2014 |
| Butyric acid | 1000 | 11.35 | 1.82 | *Rhodopseudomonas palustris* WP3-5 | 63.9 | Lo et al., 2011 |
