# Supplementary material for: Photo-hydrogen and lipid production from lactate, acetate, butyrate, and sugar manufacturing wastewater with an alternative nitrogen source by Rhodobacter sp. KKU-PS1
Source: PeerJ. 2019 Apr 4;7:e6653. doi: 10.7717/peerj.6653 (PMC6451836; doi:10.7717/peerj.6653)
Supplement: Table S2 [file peerj-07-6653-s002.docx]

| Nitrogen source | Carbon source | Concentration | H_max_ | R_m_ | H_2_ Yield | SCE | Substrate degradation | Biomass Concentration | Final pH |
| --- | --- | --- | --- | --- | --- | --- | --- | --- | --- |
|  |  | (mM) | (mL H_2_/L) | (mL H_2_/L.h) | (mol H_2_/mol substrate) | (%) | (%) | (g_CDW_/L) |  |
| Waste from crystallizing process in the monosodium glutamate industry (Aji-L) | Lactate | 5-1 |  |  |  |  |  | 0.72 | 7.17 |
|  |  | 5-2 | 149.78 ± 4.31 | 2.08 ± 0.14 | 3.27 ± 0.16 | 54.42 ± 2.60 | 100.00 ± 0.00 | 0.74 | 7.15 |
|  |  | 5-3 |  |  |  |  |  | 0.71 | 7.16 |
|  |  | 15-1 |  |  |  |  |  | 0.82 | 7.20 |
|  |  | 15-2 | 1117.50 ± 36.12 | 5.43 ± 0.35 | 3.70 ± 0.03 | 61.67 ± 0.45 | 100.00 ± 0.00 | 0.77 | 7.22 |
|  |  | 15-3 |  |  |  |  |  | 0.77 | 7.22 |
|  |  | 20-1 |  |  |  |  |  | 0.85 | 7.16 |
|  |  | 20-2 | 1465.72 ± 33.14 | 4.91 ± 0.21 | 3.71 ± 0.01 | 61.84 ± 0.17 | 100.00 ± 0.00 | 0.83 | 7.17 |
|  |  | 20-3 |  |  |  |  |  | 0.84 | 7.14 |
|  |  | 25-1 |  |  |  |  |  | 0.91 | 7.09 |
|  |  | 25-2 | 1727.35 ± 24.66 | 4.95 ± 0.16 | 3.44 ± 0.01 | 57.33 ± 0.12 | 100.00 ± 0.00 | 0.95 | 7.11 |
|  |  | 25-3 |  |  |  |  |  | 0.98 | 7.08 |
|  |  | 30-1 |  |  |  |  |  | 1.01 | 7.11 |
|  |  | 30-2 | 1978.00 ± 47.68 | 4.14 ± 0.16 | 3.31 ± 0.02 | 55.20 ± 0.40 | 100.00 ± 0.00 | 1.01 | 7.11 |
|  |  | 30-3 |  |  |  |  |  | 1.00 | 7.14 |
|  | Acetate | 10-1 |  |  |  |  |  | 0.74 | 7.26 |
|  |  | 10-2 | 235.55 ± 5.73 | 3.99 ± 0.43 | 1.49 ± 0.03 | 37.26 ± 0.70 | 100.00 ± 0.00 | 0.78 | 7.22 |
|  |  | 10-3 |  |  |  |  |  | 1.01 | 7.24 |
|  |  | 20-1 |  |  |  |  |  | 1.09 | 7.26 |
|  |  | 20-2 | 486.23 ± 7.96 | 3.80 ± 0.21 | 1.40 ± 0.02 | 35.05 ± 0.60 | 100.00 ± 0.00 | 1.13 | 7.29 |
|  |  | 20-3 |  |  |  |  |  | 1.05 | 7.24 |
|  |  | 30-1 |  |  |  |  |  | 1.57 | 7.66 |
|  |  | 30-2 | 541.26 ± 4.93 | 3.01 ± 0.07 | 1.04 ± 0.01 | 26.01 ± 0.15 | 100.00 ± 0.00 | 1.55 | 7.66 |
|  |  | 30-3 |  |  |  |  |  | 1.52 | 7.65 |
|  |  | 40-1 |  |  |  |  |  | 1.92 | 7.85 |
|  |  | 40-2 | 754.22 ± 16.06 | 3.12 ± 0.14 | 1.09 ± 0.02 | 27.31 ± 0.59 | 100.00 ± 0.00 | 1.91 | 7.90 |
|  |  | 40-3 |  |  |  |  |  | 1.90 | 7.90 |
|  |  | 5-1 |  |  |  |  |  | 0.85 | 7.34 |
|  |  | 5-2 | 434.39 ± 9.91 | 5.70 ± 0.43 | 4.81 ± 0.06 | 48.13 ± 0.59 | 100.00 ± 0.00 | 0.88 | 7.23 |
|  |  | 5-3 |  |  |  |  |  | 0.86 | 7.23 |
|  |  | 15-1 |  |  |  |  |  | 1.12 | 7.14 |
|  |  | 15-2 | 1352.68 ± 25.43 | 3.98 ± 0.20 | 4.57 ± 0.03 | 45.69 ± 0.34 | 97.08 ± 0.02 | 1.11 | 7.18 |
|  |  | 15-3 |  |  |  |  |  | 1.09 | 7.11 |
|  |  | 30-1 |  |  |  |  |  | 1.00 | 7.09 |
|  |  | 30-2 | 502.03 ± 5.94 | 2.38 ± 0.11 | 2.37 ± 0.15 | 23.74 ± 1.51 | 34.98 ± 1.89 | 1.01 | 7.17 |
|  |  | 30-3 |  |  |  |  |  | 0.99 | 7.20 |
|  |  | 45-1 |  |  |  |  |  | 0.95 | 7.05 |
|  |  | 45-2 | 338.67 ± 15.97 | 2.29 ± 0.30 | 2.14 ± 0.86 | 21.44 ± 1.92 | 17.49 ± 2.79 | 0.97 | 7.08 |
|  |  | 45-3 |  |  |  |  |  | 0.93 | 7.02 |
|  |  | 60-1 |  |  |  |  |  | 1.04 | 7.12 |
|  |  | 60-2 | 187.21 ± 4.08 | 1.79 ± 0.14 | 1.17 ± 1.72 | 11.66 ± 2.14 | 13.09 ± 1.27 | 0.85 | 7.07 |
|  |  | 60-3 |  |  |  |  |  | 0.86 | 7.10 |
|  |  | 75-1 |  |  |  |  |  | 1.01 | 7.29 |
|  |  | 75-2 | 0 | 0 | 0 | 0 | 3.84 ± 0.83 | 0.99 | 7.22 |
|  |  | 75-3 |  |  |  |  |  | 0.96 | 7.21 |
