# Supplementary material for: Photo-hydrogen and lipid production from lactate, acetate, butyrate, and sugar manufacturing wastewater with an alternative nitrogen source by Rhodobacter sp. KKU-PS1
Source: PeerJ. 2019 Apr 4;7:e6653. doi: 10.7717/peerj.6653 (PMC6451836; doi:10.7717/peerj.6653)
Supplement: Table S3 [file peerj-07-6653-s003.docx]

| Nitrogen source | Carbon source | Concentration | H_max_ | R_m_ | H_2_ Yield | SCE | Substrate degradation | Biomass Concentration | Final pH |
| --- | --- | --- | --- | --- | --- | --- | --- | --- | --- |
|  |  | (mM) | (mL H_2_/L) | (mL H_2_/L.h) | (mol H_2_/mol substrate) | (%) | (%) | (g_CDW_/L) |  |
| Monosodium glutamate | Lactate | 5-1 |  |  |  |  |  | 0.64 | 7.09 |
|  |  | 5-2 | 151.34 ± 19.02 | 2.10 ± 0.17 | 3.07 ± 0.07 | 51.15 ± 1.14 | 100.00 ± 0.00 | 0.59 | 7.11 |
|  |  | 5-3 |  |  |  |  |  | 0.74 | 7.10 |
|  |  | 15-1 |  |  |  |  |  | 0.75 | 7.07 |
|  |  | 15-2 | 1032.86 ± 42.78 | 3.14 ± 0.13 | 3.55 ± 0.03 | 59.17 ± 0.44 | 100.00 ± 0.00 | 0.73 | 7.08 |
|  |  | 15-3 |  |  |  |  |  | 0.81 | 7.17 |
|  |  | 20-1 |  |  |  |  |  | 0.82 | 7.10 |
|  |  | 20-2 | 1053.93 ± 17.63 | 3.49 ± 0.09 | 2.77 ± 0.03 | 46.15 ± 0.53 | 100.00 ± 0.00 | 0.87 | 7.12 |
|  |  | 20-3 |  |  |  |  |  | 0.77 | 7.10 |
|  |  | 25-1 |  |  |  |  |  | 0.82 | 7.13 |
|  |  | 25-2 | 1797.03 ± 35.45 | 3.78 ± 0.12 | 3.68 ± 0.01 | 61.25 ± 0.14 | 100.00 ± 0.00 | 0.81 | 7.15 |
|  |  | 25-3 |  |  |  |  |  | 0.83 | 7.13 |
|  |  | 30-1 |  |  |  |  |  | 0.96 | 7.16 |
|  |  | 30-2 | 1897.31 ± 34.75 | 3.56 ± 0.09 | 3.32 ± 0.75 | 55.37 ± 0.34 | 100.00 ± 0.00 | 1.11 | 7.19 |
|  |  | 30-3 |  |  |  |  |  | 0.94 | 7.18 |
|  | Acetate | 10-1 |  |  |  |  |  | 0.58 | 7.13 |
|  |  | 10-2 | 306.47 ± 8.27 | 3.35 ± 0.25 | 1.99 ± 0.02 | 49.71 ± 0.48 | 100.00 ± 0.00 | 0.58 | 7.14 |
|  |  | 10-3 |  |  |  |  |  | 0.59 | 7.17 |
|  |  | 20-1 |  |  |  |  |  | 0.89 | 7.23 |
|  |  | 20-2 | 589.68 ± 24.51 | 3.22 ± 0.21 | 1.81 ± 0.02 | 45.28 ± 0.42 | 100.00 ± 0.00 | 0.90 | 7.23 |
|  |  | 20-3 |  |  |  |  |  | 0.91 | 7.23 |
|  |  | 30-1 |  |  |  |  |  | 1.53 | 7.76 |
|  |  | 30-2 | 774.89 ± 21.04 | 2.28 ± 0.08 | 1.22 ± 0.02 | 30.39 ± 0.58 | 100.00 ± 0.00 | 1.50 | 7.75 |
|  |  | 30-3 |  |  |  |  |  | 1.51 | 7.68 |
|  |  | 40-1 |  |  |  |  |  | 1.57 | 7.76 |
|  |  | 40-2 | 794.92 ± 13.91 | 2.49 ± 0.10 | 1.20 ± 0.01 | 29.96 ± 0.27 | 100.00 ± 0.00 | 1.46 | 7.77 |
|  |  | 40-3 |  |  |  |  |  | 1.58 | 7.75 |
|  | Butyate | 5-1 |  |  |  |  |  | 0.77 | 7.19 |
|  |  | 5-2 | 532.21 ± 16.75 | 5.01 ± 0.41 | 6.05 ± 0.08 | 60.49 ± 0.77 | 100.00 ± 0.00 | 0.74 | 7.22 |
|  |  | 5-3 |  |  |  |  |  | 0.82 | 7.22 |
|  |  | 15-1 |  |  |  |  |  | 0.80 | 7.18 |
|  |  | 15-2 | 733.85 ± 9.04 | 3.27 ± 0.13 | 4.02 ± 0.35 | 40.20 ± 0.90 | 60.52 ± 0.67 | 0.79 | 7.18 |
|  |  | 15-3 |  |  |  |  |  | 0.81 | 7.16 |
|  |  | 30-1 |  |  |  |  |  | 0.83 | 7.21 |
|  |  | 30-2 | 437.37 ± 23.11 | 3.04 ± 0.47 | 3.35 ± 0.32 | 32.57 ± 3.19 | 29.93 ± 4.53 | 0.90 | 7.24 |
|  |  | 30-3 |  |  |  |  |  | 0.76 | 7.25 |
|  |  | 45-1 |  |  |  |  |  | 0.82 | 7.24 |
|  |  | 45-2 | 199.89 ± 6.09 | 2.92 ± 0.41 | 1.21 ± 0.29 | 12.14 ± 1.41 | 19.07 ± 1.69 | 0.81 | 7.25 |
|  |  | 45-3 |  |  |  |  |  | 0.82 | 7.26 |
|  |  | 60-1 |  |  |  |  |  | 0.86 | 7.32 |
|  |  | 60-2 | 240.00 ± 9.42 | 2.04 ± 0.14 | 1.48 ± 0.42 | 14.74 ± 2.42 | 14.99 ± 2.48 | 0.85 | 7.28 |
|  |  | 60-3 |  |  |  |  |  | 0.87 | 7.30 |
|  |  | 75-1 |  |  |  |  |  | 0.69 | 7.23 |
|  |  | 75-2 | 0 | 0 | 0 | 0 | 5.68 ± 0.85 | 0.92 | 7.29 |
|  |  | 75-3 |  |  |  |  |  | 0.73 | 7.24 |
