# Supplementary material for: Photo-hydrogen and lipid production from lactate, acetate, butyrate, and sugar manufacturing wastewater with an alternative nitrogen source by Rhodobacter sp. KKU-PS1
Source: PeerJ. 2019 Apr 4;7:e6653. doi: 10.7717/peerj.6653 (PMC6451836; doi:10.7717/peerj.6653)
Supplement: Table S4 [file peerj-07-6653-s004.docx]

| Carbon sources | H_max_ | R_m_ | HY | Substrate degradation (%) | Biomass | Final pH |
| --- | --- | --- | --- | --- | --- | --- |
|  | (mL H_2_/L) | (mL H_2_/L.h) | (mol H_2_/mol _substrate_) |  | Concentration (g_CDW_/L) |  |
| e-SMW | 1888 ± 138 | 3.76 ± 0.05 | 2.58 ± 0.12 | 74.86 ± 0.91 | 2.54 ± 0.25 | 7.66 ± 0.01 |
| SMW | 1672 ± 99 | 4.62 ± 0.08 | 1.92 ± 0.67 | 65.08 ± 0.76 | 2.23 ± 0.11 | 7.48 ± 0.01 |
| H_max_: maximum cumulative hydrogen production, R_m_: maximum hydrogen production rate, HY: hydrogen yield | | | | | | |
